# Supplementary material for: Revisiting the cognitive and behavioral aspects of loneliness: Insights from different measurement approaches
Source: PLoS One. 2025 Apr 28;20(4):e0321931. doi: 10.1371/journal.pone.0321931 (PMC12036861; doi:10.1371/journal.pone.0321931)
Supplement: Attachment — Submitted filename: Response to Reviewers.docx [file pone.0321931.s002.docx]

## Point-by-point response

Comment from the authors:

We thank all reviewers very much for their time and their valuable and thoughtful comments on our manuscript. All revisions are highlighted in the manuscript. We wholeheartedly believe that the suggestions and our respective changes improved the manuscript significantly.

After implementing the comments we streamlined several parts of the manuscript and rearranged the results section to achieve a more logical structure. Further, we adjusted Table 4 by replacing the sum scores with the means and standard deviations of the lonely and non-lonely groups in terms of cognitive and behavioral aspects, since we believe it would be more intuitive information for the reader.

### Reviewer #1:

Abstract:

Comment 1:

- This is just my personal opinion, but “ancova” as a keyword seems strange to me. It's not a specific modeling approach that people might be looking for, nor is it a content-related keyword.
- Response: We appreciate this suggestion and agree that "ancova" may not be an intuitive keyword for this manuscript. We have replaced it with “social cognition” - a more content-related term.

Introduction:

Comment 2:

- In the research I was involved in, we looked at the fact that loneliness should be differentiated at the state and trait level. This aspect could fit well in the introduction (only if the authors consider it relevant).

Gründahl, M., Weiß, M., Maier, L., Hewig, J., Deckert, J., & Hein, G. (2022). Construction and validation of a scale to measure loneliness and isolation during social distancing and its effect on mental health. Frontiers Psychiatry, 13, 798596.

- Response: Thank you for the suggestion. We appreciated looking into this research and acknowledge the importance of differentiating between state and trait loneliness. However, we refrained from introducing more terms regarding different aspects of loneliness but still incorporated this valuable paper in this section in the introduction:

*“However, some scholars define maladaptive forms of loneliness based on frequency [e.g., 40], others based on distress associated with loneliness [e.g., 37], and others based on chronicity [e.g., 41]. To counter this heterogeneity, recent research has increasingly emphasized the importance of assessing loneliness as a multidimensional construct, incorporating different aspects [e.g., 31, 42]. These efforts align with the broader need to refine loneliness measurement and account for its diverse manifestations.”*

Comment 3:

- Overall, I really liked the introduction very much. If the authors could think about how to tighten it up a bit, I think a lot of people might enjoy reading it. The rationale for the approach is well explained. It's a bit sad that the authors did not pre-register their study.
- Response: We appreciate the positive feedback on the introduction and have revised it to enhance conciseness while maintaining clarity (see highlighted sections in the manuscript). Regarding pre-registration, we agree and acknowledge this limitation and now mention it explicitly in the limitations section:

  *“Finally, this study it should be mentioned that this study was not pre-registered, which limits transparency in hypothesis testing.”*

Methods:

Comment 4:

- Please provide further information on the survey: How many participants dropped out of the study? Was there a data-related quality/exclusion criterion (e.g., straightlining)? Were the questionnaires presented in random order or always in the same order (which order?)?
- Response: We appreciate the suggestion to provide additional details about the survey procedure and data quality control. We have now included information regarding participant dropout rates, exclusion criteria, and questionnaire presentation order in the Methods section:

*“A total of 1,389 individuals initially accessed the online survey. Of these, 553 participants (39.8%) did not finish the survey and were excluded from the final sample. To ensure data integrity, bogus items were implemented in the survey, where another 23 participants were excluded. Two participants who reported being under the age of 18 were also excluded. Finally, we used complete-case analysis, leading to the exclusion of an additional 21 participants. The final sample consisted of 790 German-speaking adult participants.”*

*“All questionnaires were presented in a fixed order to maintain consistency. The order was as follows: (1) demographic information, (2) loneliness measures, (3) cognitive and behavioral variables, and (4) psychopathological*.”

Results:

Comment 5:

- As the authors state in their introduction, loneliness can also maintain or further reinforce maladaptive cognitive and behavioral patterns. Since this study is cross-sectional, the direction is not clear. I wondered if adding three logistic regressions (one for each division method) using all the outcomes from the ancovas as predictors could also account for this aspect? This is by no means mandatory, it was just an idea that could also be argued against.
- Response: Thank you for the interesting suggestion to conduct additional logistic regressions to explore whether the cognitive and behavioral variables predict loneliness classifications. While we acknowledge the importance of examining potential directional influences, we decided not to include these additional analyses for several reasons:
  - Our primary goal is to test the cognitive model of loneliness, not necessarily to determine the predictive strength of each component for loneliness classification. While predictive models can be valuable, our study is more concerned with exploring group differences based on loneliness dimensions, aligning with prior literature that takes a categorical approach.
  - Since our study is cross-sectional, even logistic regressions would not establish causal directionality. While they could identify associations between cognitive/behavioral patterns and loneliness classifications, they would not clarify whether these variables drive loneliness or result from it. Without a longitudinal or experimental design, directionality remains speculative.
  - Adding logistic regressions would increase statistical complexity without necessarily improving practical interpretability. Given the already comprehensive results presented, additional models could make the paper more cumbersome to read

Thus, while we recognize the merit of the reviewer’s suggestion, we opted to maintain our current analytic approach to keep the focus on group comparisons rather than predictive modeling. We acknowledge the study’s cross-sectional limitations and rewrote the first limitations section to acknowledge the suggestion and note that longitudinal studies would be better suited to address causality and directionality concerns.

“*First, while our findings indicate significant differences in cognitive and behavioral processes across different loneliness classifications, the cross-sectional design does not allow for conclusions about causality. Future research should employ longitudinal designs to disentangle whether cognitive biases and behavioral tendencies contribute to loneliness persistence or emerge as a consequence of prolonged loneliness.”*

Limitations:

Comment 6:

- I don't want to start an unrelated Covid debate here. But the question about the last 24 months in 2021/2022 could have led to a distorted self-assessment of chronic loneliness, as many people may have thought about lockdowns, etc. This could also (partly) explain the relatively high prevalence of chronic loneliness.
- Response: We appreciate the concern regarding the potential influence of COVID-19 on self-reports of chronic loneliness. The 24-month timeframe (2021/2022) coincided with the pandemic, raising the possibility that some individuals might have provided distorted perceptions of the duration of loneliness due to social distancing.

However, evidence suggests that the pandemic did not uniformly increase loneliness across all populations. For example, a meta-analysis by Ernst et al. (2022) found small but significant increases in loneliness prevalence during the pandemic. However, these increases varied across studies and populations, with some reporting no changes or even decreases in loneliness. This might suggest that while COVID-19 may have amplified loneliness for some, it is unlikely to be the sole explanatory factor for chronic loneliness prevalence.

Furthermore, chronic loneliness is not solely dependent on social isolation but is also shaped by stable individual factors, cognitive patterns, and perceived social disconnection. To acknowledge this potential bias, we have added a discussion to the Limitations section:

*“Ninth, this study's assessment of chronic loneliness covered the past 24 months, a period that overlapped with the COVID-19 pandemic. This raises the possibility that some participants may have perceived the duration of their loneliness as chronic due to social distancing and lockdowns, even if it could have been primarily situational. However, research suggests that while overall loneliness increased during the pandemic, the effects were small [6]. Given that loneliness is influenced by stable cognitive and emotional factors rather than just external circumstances, the extent of this bias remains unclear. Future research should compare post-pandemic data to determine whether chronic loneliness rates have returned to pre-pandemic levels.”*

### Reviewer #2:

Comment 1:

- Lines 30-32: The statement on the division of the sample into "lonely and not lonely" individuals could be clarified. I suggest adding a more detailed description of the methodology used to distinguish between the different groups, specifying the criteria for each measure more clearly.
- Response: We are thankful for this suggestion. Given the space limitations of the abstract, we aim to provide a concise summary rather than a detailed methodological explanation. However, we have slightly clarified the wording to ensure the classification criteria are easily understood. A full description of the methodology is available in the Methods section.

*“We classified 790 German-speaking adults (M_Age_ = 31.86 (12.48), 81% female) as lonely or not lonely based on three dimensions - loneliness frequency, distress, and chronicity - and tested for group differences regarding cognitive and behavioral aspects, as proposed by the cognitive model of loneliness, while controlling for depressive and social anxiety symptoms.”*

Comment 2:

- Lines 49-51: The control of depressive and social anxiety symptoms is mentioned. It would be helpful to expand this section by providing more details on the methods and tools used to control for these factors.
- Response: Thank you for your comment. We agree that providing more details on the methods used to control for depressive and social anxiety symptoms enhances clarity. To address this, we have expanded the relevant section to specify that depressive symptoms were assessed using the Patient Health Questionnaire-9 (PHQ-9), and social anxiety symptoms were measured using the Social Interaction Anxiety Scale-6 (SIAS-6). Both measures were included as covariates in our analyses to account for their potential influence on cognitive and behavioral differences in loneliness. This information has now been incorporated into the manuscript to ensure transparency regarding our methodological approach.

*“Sixth, we performed three (for each classification method) two-sided ANCOVA to test group differences regarding interpretation bias, rejection sensitivity, social avoidance behavior, distress disclosure, self-esteem, and avoidance goal intensity while controlling for depressive (PHQ-9) and social anxiety symptoms (SIAS-6) by including them as continuous covariates. This approach allowed us to isolate the specific effects of loneliness facets on cognitive and behavioral variables and ensured that observed differences are not solely driven by underlying psychopathology.”*

Comment 3:

- Lines 121-125: The definition of "chronic loneliness" could benefit from further elaboration, explaining the choice of the 24-month period as a threshold for chronicity, perhaps integrating additional studies that support this choice.
- Response: Thank you for your suggestion. We have clarified our operationalization of chronic loneliness in the method section. We based our definition on Young (1982), who distinguished chronic loneliness from transient loneliness by defining it as a state in which an individual has experienced loneliness for two or more years. However, while we followed Young’s definition, previous studies have also used the two-year threshold to define chronic loneliness, either through self-reported duration in cross-sectional studies (George, 1984; Gerson & Perlman, 1979; Hojat, 1983) or primarily in longitudinal designs where loneliness had to be present at two consecutive time points to be classified as chronic (e.g., Lim et al., 2023; Martin-María et al., 2021; Theeke, 2010). Due to the cross-sectional design, we used the former method and adapted it by incorporating the direct single-item with the threshold of feeling lonely at least “quiet often”. We contextualized this more in the introduction and the method sections:

Introduction:

“*In the context of specifically investigating chronic loneliness, the definition of Young [43] has been used as a reference, which characterizes chronic loneliness as loneliness persisting for two years and beyond. Older cross-sectional studies relied on the self-reported duration of loneliness to operationalize chronic loneliness [44-46]. As previously mentioned, Qualter et al. [31] pointed out that most loneliness scales, which are frequently used in recent studies, typically ask participants about the frequency of their loneliness, using Likert-type scales from 'never' to 'always.’ These scales are then also applied in longitudinal studies to classify participants into 'not lonely,' 'temporary lonely,’ and 'chronically lonely' groups, where the occurrence of a certain level of loneliness at all time points determines the affiliation to one of the categories [e.g., 47, 48-52]. These study designs seem to integrate both duration and frequency aspects. However, one potential issue emerging from this is that it is unclear how well chronic loneliness is encompassed in these studies since changes in loneliness between the time points were not captured.”*

Methods:

*“We then created a dichotomous variable (1 = lonely and 0 = non-lonely) with a cut-off value of 24 months in accordance with the definition by Young [43], which has been used in previous cross-sectional [44-46] and longitudinal studies [e.g., 47, 48-52].”*

Comment 4:

- Lines 206-210: The results regarding the agreement between the three loneliness measures (frequency, distress, chronicity) could be supported by explanatory graphs that visually illustrate the percentage of agreement and disagreement between the different measures.
- Response: Thank you for your suggestion. We have now added a bar chart that visually illustrates the percentage agreement between the different loneliness measures (frequency, distress, and chronicity). We believe that this addition makes the results more accessible and enhances the interpretation of our findings.

Comment 5:

- Lines 255-265: The interpretation of the correlation between loneliness and social avoidance behavior could be enriched by discussing possible psychological mechanisms that might explain this link.
- Response: Thank you for your suggestion. We have expanded the discussion of the link between loneliness and social avoidance behavior by integrating potential psychological mechanisms that may explain this relationship. Specifically, we now emphasize how interpretation bias and rejection sensitivity may contribute to social avoidance, which in turn limits opportunities for social connection and exacerbates loneliness.

*“Lonely individuals tend to anticipate rejection, leading them to be more vigilant for social threats [79, 80], which is theorized to promote social withdrawal [10, 12]. Accordingly, our results show that loneliness comes with increased social avoidance behavior. This finding is in line with studies showing that lonely individuals are more likely to engage in behaviors that avoid social interactions [80, 81]. Notably, rejection sensitivity has been shown to predict social avoidance behavior [80] and thus may potentially exacerbate feelings of loneliness.”*

Comment 6:

- Lines 350-355: I suggest a broader discussion of the study's limitations. Although depressive and social anxiety symptoms were included, there may be other confounding factors, such as social networks or the quality of interpersonal relationships, that deserve to be mentioned as potential influences not accounted for.
- Response: Thank you for pointing this out. While we controlled for depressive and social anxiety symptoms, we recognize that interpersonal relationship quality and broader social context could also play a crucial role in shaping loneliness and its cognitive-behavioral correlates. These factors should be considered in future research to provide a more comprehensive understanding of the mechanisms underlying loneliness. We have already acknowledged other potential confounding variables in our limitations but rephrased them due to your comment:

“*Sixth, we also did not account for all potential confounding variables. While controlling for depressive and social anxiety symptoms, factors like personality traits, quality of interpersonal relationships, and recent life events were not considered. Including these factors in future studies would strengthen the findings.“*

Comment 7:

- Lines 400-405: It would be helpful to include a dedicated section on the study’s limitations. For example, the cross-sectional nature of the study design might limit causal conclusions. Additionally, the influence of potential self-selection bias in the sample collection should be considered, given that most participants were women and the average age was relatively young.
- Response: Thank you for your valuable suggestion. We agree with the importance of acknowledging the limitations of our study and have, therefore, already discussed the cross-sectional design and included suggestions from Reviewer 1. However, we did mention the self-selection bias regarding our prevalences in the discussion (Section 4.1.) but not in the limitation section. We included this point in the following section in the manuscript:

*“First, while our findings indicate significant differences in cognitive and behavioral processes across different loneliness classifications, the cross-sectional design does not allow for conclusions about causality. Future research should employ longitudinal designs to disentangle whether cognitive biases and behavioral tendencies contribute to loneliness persistence or emerge as a consequence of prolonged loneliness. Second, a potential limitation of this study is the presence of self-selection bias, which may have influenced the characteristics of our sample. Given that our study was explicitly advertised as a study on loneliness, it is possible that individuals who experience loneliness were more motivated to participate, leading to an overrepresentation of lonely individuals. This may have inflated prevalence rates. Third, the sample was predominantly female (81%), which limits generalizability. While a previous meta-analysis has found that gender differences in loneliness are generally small [94], a newer study reported higher levels of loneliness in men than women [95]. Fourth, our sample was relatively young. While this limits the generalizability of our findings to older or adolescent populations, prior research suggests that key cognitive and behavioral mechanisms underlying loneliness - such as interpretation bias and social avoidance - are relevant across age groups [10, 13]. Nonetheless, future studies should investigate whether the relationships observed in our study differ across developmental stages.”*

Comment 8:

- Lastly, I recommend expanding the clinical implications section by providing more concrete examples of how interventions can be adapted to the different dimensions of loneliness identified.
- Response: Thank you for your suggestion. We have expanded the clinical implications section to provide more concrete examples of how interventions can be adapted to the different dimensions of loneliness. Specifically, we now highlight that one-size-fits-all approaches may not be optimal, as different facets of loneliness—frequency, distress, and chronicity—may require distinct intervention strategies. Drawing on Käll et al. (2020), we suggest that interventions should be modular and tailored to address specific cognitive, emotional, and behavioral mechanisms associated with each aspect of loneliness. Additionally, we acknowledge that these are preliminary recommendations and future research is needed to systematically investigate how intervention strategies can be adapted to these distinct loneliness dimensions.

*“Similarly, Käll et al. [39] argue against “one-size fits all” approaches and suggest modular interventions that address specific cognitive, emotional, and behavioral mechanisms of loneliness may be more effective. For instance, those experiencing frequent loneliness might benefit from cognitive restructuring, while individuals with high distress could focus on emotional regulation, and those with chronic loneliness may require interventions targeting long-term avoidance patterns and social skills training. However, future research is needed to systematically examine how different intervention strategies can be tailored to distinct dimensions of loneliness.”*

Comment 9:

- Regarding the data analysis and results, the statistical methods used are appropriate and rigorous, with suitable controls for depressive and social anxiety symptoms. However, I would recommend providing more details on the adjusted p values using the Benjamini-Hochberg method and on interaction effects between the cognitive and behavioral variables.
- Response: We appreciate the suggestion to provide more details on the adjusted p-values and interaction effects. We expanded the statistical analyses section with a short elaboration on the Benjamini-Hochberg correction.

As for the interaction effects, given the cross-sectional nature of our study, such analyses would not allow for meaningful conclusions about dynamic processes between cognitive and behavioral variables. Interaction effects imply moderation, which typically requires temporal precedence or experimental manipulation to establish meaningful relationships (Frazier et al., 2004). Since loneliness and its associated factors are theorized to interact in bidirectional feedback loops over time (Cacioppo & Hawkley, 2009; Qualter et al., 2015), a longitudinal design would be more appropriate for investigating these interactions. Future research should explore these mechanisms using longitudinal or experimental approaches.

“*The Benjamini-Hochberg correction controls the false discovery rate by ranking p-values in ascending order and adjusting them based on their rank and the total number of tests.*”

Comment 10:

- No specific sections need to be eliminated, but I suggest streamlining some parts of the discussion to reduce repetition and make the text more fluid.
- Response: We appreciate the suggestion to streamline the discussion. In response to Comment 5, we have already revised this section to improve clarity, reduce redundancy, and better integrate the results. This revision enhances the flow of the discussion while maintaining the key interpretations of our findings.

Comment 11:

- In conclusion, the work presented is of great scientific value and significantly contributes to the existing literature. I hope these suggestions will help further refine the manuscript. Once again, thank you for entrusting me with the review of this important contribution.
  I would kindly suggest that you include a reference to the following article in your manuscript:

Diotaiuti, P., Valente, G., Mancone, S., Grambone, A., & Chirico, A. (2021). Metric goodness and measurement invariance of the Italian brief version of Interpersonal Reactivity Index: A study with young adults. Frontiers in Psychology, 12, Article 773363. <https://doi.org/10.3389/fpsyg.2021.773363>.
This article is highly relevant for your discussion on measurement tools, particularly when addressing the methodological aspects related to the validation and reliability of psychological scales. I recommend citing this work in the section where you discuss the psychometric properties of the tools used to measure loneliness, specifically around Lines 150-160, where you explore the reliability and validity of different scales. The reference could enrich your discussion on the robustness of measurement instruments across different cultural and demographic groups.

- Response: We appreciate the very positive evaluation of our work and the suggestion to include the reference by Diotaiuti et al. (2021). While the study provides valuable insights into the measurement invariance and psychometric validation of the Interpersonal Reactivity Index, we do not focus on empathy or the IRI in our study. As a result, we do not see a direct connection to our discussion of loneliness measures. However, we acknowledge the broader relevance of psychometric considerations and appreciate the recommendation.

### Reviewer #3:

Comment 1:

- I suggest the authors to directly indicate the three definitions of loneliness and the study participants in the title, to be strict.
- Response: We appreciate the suggestion. However, we believe that including all three facets of loneliness and specifying the study participants in the title would make it overly long and potentially less clear. Even though our sample is rather young, it does include a wider age range, which makes it difficult to describe the sample in the title. Instead, we aim for a concise and engaging title that accurately reflects the study’s focus. We will ensure that the abstract and introduction clearly outline the definitions of loneliness used and the characteristics of our sample.

Comment 2:

- In the introduction, when describing the negative mental and physical health outcomes of enduring loneliness, the authors must be aware of the limited availability of evidence on some consequences of persistent loneliness (PMID: 24550354, PMID: 26905049). I suggest the authors to revise the sentence “While nearly everyone experiences loneliness at some point in their life, for some, it can become an enduring condition with significant negative implications for mental and physical health and even increased mortality, elevating loneliness as a global health priority”.
- Response: We appreciate the reviewer’s comment and agree that the evidence for some long-term health consequences of loneliness is still developing. To address this, we have revised the sentence to more accurately reflect the current state of research:

“*While nearly everyone experiences loneliness at some point in their life, for some, it can become an enduring condition with potentially negative implications for mental and physical health and even increased mortality [see for reviews 2, 3, 4], elevating loneliness as a global health priority [5].”*

Comment 3:

- In the part of assessing loneliness, please consider to review the De Jong-Gierveld Loneliness Scale and explain why the authors did not use it.
- Response: We appreciate the reviewer’s suggestion. The De Jong-Gierveld Loneliness Scale (also called Rasch-Type Loneliness Scale) is indeed a well-established measure of loneliness, particularly in capturing social and emotional loneliness. However, we opted not to use this scale because our study focused on distinguishing loneliness facets in terms of frequency, distress, and chronicity. In contrast to the UCLA-LS, the De Jong-Gierveld scale does not asses the frequency of loneliness. Therefore, we used measures that align more directly with our conceptual framework and research questions.

Comment 4:

- In the methodology, please consider the poor representativeness of the study sample. The authors need to be aware of the different constructs of loneliness in adolescents, adults, and older adults.
- Response: We appreciate your concern regarding age-related differences in loneliness. Qualter et al. (2015) highlight that loneliness evolves across the lifespan, but they also suggest that key cognitive and behavioral mechanisms - such as interpretation bias and social avoidance - are relevant across age groups. Our study focuses on adults (M = 31.86 years, SD = 12.48, range = 18–90) and does not claim direct generalizability to adolescents or older adults. However, our findings align with prior research (e.g., Spithoven et al., 2017), showing that cognitive biases and social avoidance could play a role in maintaining loneliness in adult populations, even when controlling for depressive and social anxiety symptoms.

We expanded the paragraph in the limitation section regarding age:

“*Fourth, our sample was relatively young. While this limits the generalizability of our findings to older or adolescent populations, prior research suggests that key cognitive and behavioral mechanisms underlying loneliness - such as interpretation bias and social avoidance - are relevant across age groups [10, 13]. Nonetheless, future studies should investigate whether the relationships observed in our study differ across developmental stages.”*

Comment 5:

- The online survey was conducted during the COVID-19 pandemic but the authors did not have comments on this special social context (PMID: 37562972). My concern the generalizability of the current findings.
- Response: We appreciate your concern regarding the potential impact of the COVID-19 pandemic on our findings. As Reviewer 1 also noted, our study assessed loneliness chronicity over the past 24 months, which overlapped with the pandemic. To address this, we have already included a section discussing the potential influence of pandemic-related social restrictions on participants' self-reported loneliness. Specifically, we acknowledge that some participants may have perceived their loneliness as chronic due to social distancing and lockdowns, even if it was primarily situational. However, we also note that research suggests that while overall loneliness increased during the pandemic, the effects were relatively small [86]. Given that loneliness is argued and shown to be affected by stable cognitive and emotional factors rather than just external circumstances, we argue that the extent of pandemic-related bias remains unclear.

To further address concerns about generalizability, we emphasize the importance of future research examining whether similar patterns emerge in post-pandemic data. This will help clarify the extent to which our findings reflect broader, enduring mechanisms of loneliness rather than pandemic-specific effects.

“*Ninth, this study's assessment of chronic loneliness covered the past 24 months, a period that overlapped with the COVID-19 pandemic. This raises the possibility that some participants may have perceived the duration of their loneliness as chronic due to social distancing and lockdowns, even if it could have been primarily situational. However, research suggests that while overall loneliness increased during the pandemic, the effects were small [6]. Given that loneliness is influenced by stable cognitive and emotional factors rather than just external circumstances, the extent of this bias remains unclear. Future research should compare post-pandemic data to determine whether chronic loneliness rates have returned to pre-pandemic levels.”*

### Reviewer #4:

Comment 1:

- While the authors acknowledge the cross-sectional nature of the study, additional discussion on how self-selection bias might have impacted the findings (given that participants self-identified for a loneliness study) would be beneficial. More emphasis on potential biases due to the high proportion of female participants (81%) and the relatively young sample would also strengthen the paper.
- Response: We appreciate this comment and agree that a more detailed discussion of self-selection bias and sample composition is warranted. While we already acknowledge the potential self-selection bias in the discussion section when interpreting the prevalence rates of loneliness, we have now expanded the limitations section to explicitly address how this might have influenced our findings.

“*Second, a potential limitation of this study is the presence of self-selection bias, which may have influenced the characteristics of our sample. Given that our study was explicitly advertised as a study on loneliness, it is possible that individuals who experience loneliness were more motivated to participate, leading to an overrepresentation of lonely individuals. This may have inflated prevalence rates. Third, the sample was predominantly female (81%), which limits generalizability. While a previous meta-analysis has found that gender differences in loneliness are generally small [94], a newer study reported higher levels of loneliness in men than women [95]. Fourth, our sample was relatively young. While this limits the generalizability of our findings to older or adolescent populations, prior research suggests that key cognitive and behavioral mechanisms underlying loneliness - such as interpretation bias and social avoidance - are relevant across age groups [10, 13]. Nonetheless, future studies should investigate whether the relationships observed in our study differ across developmental stages.”*

Comment 2:

- The use of a self-reported measure of chronicity (24 months) could be refined further. While grounded in previous literature, it may not fully capture the nuanced experiences of chronic loneliness and a more detailed discussion of why this specific threshold was used and its potential limitations would add clarity.
- Response: We appreciate the suggestion to further refine use of the self-reported measure of chronicity (24 months) and acknowledge its potential limitations. Following the suggestion by Reviewer 2, we have already expanded our justification for using the two-year threshold in our introduction and methods sections, citing its foundation in Young (1982) and its widespread adoption in both cross-sectional (e.g., George, 1984; Gerson & Perlman, 1979; Hojat, 1983) and longitudinal studies (e.g., Lim et al., 2023; Martin-María et al., 2021; Theeke, 2010). However, we recognize that self-reported chronicity may not fully capture the complexity of chronic loneliness. We already highlight this in the limitation section, which you will find bellow:

Introduction:

*“In the context of specifically investigating chronic loneliness, the definition of Young [43] has been used as a reference, which characterizes chronic loneliness as loneliness persisting for two years and beyond. Older cross-sectional studies relied on the self-reported duration of loneliness to operationalize chronic loneliness [44-46]. As previously mentioned, Qualter et al. [31] pointed out that most loneliness scales, which are frequently used in recent studies, typically ask participants about the frequency of their loneliness, using Likert-type scales from 'never' to 'always.’ These scales are then also applied in longitudinal studies to classify participants into 'not lonely,' 'temporary lonely,’ and 'chronically lonely' groups, where the occurrence of a certain level of loneliness at all time points determines the affiliation to one of the categories [e.g., 47, 48-52]. These study designs seem to integrate both duration and frequency aspects. However, one potential issue emerging from this is that it is unclear how well chronic loneliness is encompassed in these studies since changes in loneliness between the time points were not captured*.”

Methods:

*“We then created a dichotomous variable with a cut-off value of 24 months in accordance with the definition by Young [43], which has been used in previous cross-sectional [44-46] and longitudinal studies [e.g., 47, 48-52].”*

Limitations

"*Seventh, our operationalization of loneliness chronicity as feelings lasting at least 24 months, while grounded in previous literature, may not fully capture the nuanced experiences of chronic loneliness. More refined measures of chronicity in longitudinal studies could provide deeper insights. Eighth, even though the cut-off points were extracted from previous studies or theories, they remain somewhat arbitrary. Changing these would have also changed the results. Nevertheless, the three methods did not seem to capture the same individuals entirely."*

Comment 3:

- Although the manuscript provides a solid analysis of the cognitive and behavioral components of loneliness, the reliance on self-report questionnaires introduces possible response biases (e.g., social desirability or recall biases). Addressing the potential limitations of self-reported data and suggesting alternative or supplementary measures (such as behavioral data) would provide more balance.
- Response: We appreciate your concern regarding the potential biases associated with self-report measures, such as social desirability and recall biases. This is a well-known limitation in psychological research, particularly in studies examining subjective experiences like loneliness. As Reviewer 2 also pointed out, self-report measures inherently rely on participants' perceptions and introspection, which may not always align with objective reality. To address this, we have already acknowledged this limitation in the manuscript and added exemplary literature for objective measures:

"*Fifth, the reliance on self-report measures introduces potential response biases. Participants may have underreported or overreported their feelings due to social desirability or recall bias. Incorporating objective measures of behavioral tendencies [e.g., 96] and cognitive biases [e.g., 97] could help mitigate these biases.* "

Comment 4:

- The implications for interventions could be expanded. While the discussion touches on how different facets of loneliness could guide targeted interventions, more specific examples or suggestions (e.g., interventions tailored to chronic vs. distress-related loneliness) would enhance the practical applicability of the findings.
- Response: We appreciate the reviewer's suggestion to expand the implications for interventions by providing more specific examples of tailored approaches for different facets of loneliness. As we mentioned in the response to Reviewer 2 (Comment 8), interventions should not adopt a one-size-fits-all approach, and different aspects of loneliness may require distinct strategies. To address this, we have already revised and expanded the discussion section, incorporating more concrete intervention examples. Specifically, we speculated how interventions could be customized based on the three loneliness dimensions (frequency, distress, chronicity):

" *Similarly, Käll et al. [39] argue against “one-size fits all” approaches and suggest modular interventions that address specific cognitive, emotional, and behavioral mechanisms of loneliness may be more effective. For instance, those experiencing frequent loneliness might benefit from cognitive restructuring, while individuals with high distress could focus on emotional regulation, and those with chronic loneliness may require interventions targeting long-term avoidance patterns and social skills training. However, future research is needed to systematically examine how different intervention strategies can be tailored to distinct dimensions of loneliness."*

Abstract:

Comment 5:

- The abstract is succinct and informative but could benefit from specifying the sample characteristics (age range, gender distribution) to provide context for the generalizability of the findings.
- Response: Thank you for your suggestion to include more details on the sample characteristics in the abstract to enhance clarity and generalizability. While we aim to keep the abstract succinct, we recognize the importance of providing context for the study sample. To address this, we have revised the abstract to incorporate key demographic information about the participants:

“*We classified 790 German-speaking adults (M_Age_ = 31.86 (12.48), 81% female) as lonely or not lonely based on three dimensions - loneliness frequency, distress, and chronicity - and tested for group differences regarding cognitive and behavioral aspects, as proposed by the cognitive model of loneliness, while controlling for depressive and social anxiety symptoms.”*

Introduction:

Comment 6:

- The introduction could briefly address the global health implications of loneliness, especially its rising importance during post-pandemic times, which would make the study even more relevant.
- Response: We appreciate the suggestion to address the global health implications of loneliness in a post-pandemic context. While research has shown that loneliness increased during the COVID-19 pandemic, the overall effect size of this change was small (Ernst et al., 2022). Yet, pre-pandemic data has shown significant prevalence of loneliness (Surkalim et al., 2022). As for the post-pandemic period, to our knowledge, comprehensive meta-analyses or reviews on loneliness levels are still limited. That is why we framed the introduction as follows:

*“Loneliness is defined as a subjectively distressing emotional state characterized by a discrepancy between the desired and perceived quality or quantity of social relationships [1]. While nearly everyone experiences loneliness at some point in their life, for some, it can become an enduring condition with potentially negative implications for mental and physical health and even increased mortality [see for reviews 2, 3, 4], elevating loneliness as a global health priority [5]. While the COVID-19 pandemic led to increased loneliness, the effect size of this change was small [6]. Still, pre-pandemic data from 113 countries showed loneliness prevalence ranging from 5.3% to 12.7% across age groups [7], emphasizing the need for continued research and intervention.”*

Results:

Comment 7:

- The presentation of statistical results is thorough. However, some readers might benefit from a more intuitive explanation of Cohen's κ and rφ values, particularly in the context of agreement between the loneliness measures.
- Response: Thank you for your suggestion. We agree that a more intuitive explanation of Cohen’s *κ* and *r_φ_* values could improve accessibility for readers unfamiliar with these statistics. To address this, we have clarified their interpretation in the method and results sections by explicitly stating that different classification methods capture distinct but overlapping aspects of loneliness:

Methods:

*“[…] Cohen’s κ [72], and r_φ_. Cohen’s κ values are interpreted as follows: values ≤ 0 indicate no agreement, 0.01–0.20 indicate slight agreement, 0.21–0.40 indicate fair agreement, 0.41–0.60 indicate moderate agreement, 0.61–0.80 indicate substantial agreement and 0.81–1.00 indicate almost perfect agreement [73]. r_φ_ values range from -1 to 1, with values closer to 1 indicating a stronger positive correlation. Cohen’s κ represents the agreement between classifications beyond chance, whereas r_φ_ quantifies the strength of association between classification methods.”*

Results:

“*In sum, these results indicate that while there is some overlap between different loneliness classification methods, they are not entirely interchangeable. The varying levels of agreement across measures suggest that each method captures a distinct aspect of loneliness, with distress and chronicity showing the highest alignment and frequency showing lower agreement with the other methods.”*

Tables:

Comment 8:

- The tables are well-organized and provide essential data. Adding brief interpretative captions below some of the more complex tables (e.g., explaining what constitutes a “strong positive association” in lay terms) could make the findings more accessible to a broader readership.
- Response: We appreciate the suggestion to improve the accessibility of complex tables by adding interpretative captions. To address this, we have clarified the meaning of effect sizes in lay terms directly below Table 4. Specifically, we added a brief explanation of how to interpret *partial η²* values, indicating the strength of associations between loneliness classifications and cognitive-behavioral variables:

*“Partial η^2^ values indicate the proportion of variance explained by loneliness classification in each cognitive-behavioral variable. Values below .01 suggest a negligible effect, .01-.06 a small effect, .06-.14 a moderate effect, and above .14 a large effect.”*

### Reviewer #5:

Comment 1:

- Clarification of theoretical frameworks: the manuscript heavily leans on established theoretical frameworks. However, it lacks a critical assessment of these frameworks. For instance, while the text acknowledges the adaptive aspects of loneliness, it does not thoroughly explore how this adaptive nature contrasts with the maladaptive outcomes. furthermore, a more nuanced discussion regarding the interplay between adaptive and maladaptive loneliness would enhance the theoretical grounding of the study.
- Response: Thank you for your suggestion. We tried to clarify the distinction between adaptive and maladaptive loneliness in the introduction, emphasizing how loneliness can initially serve an adaptive function but may become maladaptive when persistent and that individuals can escape maladaptive tendencies, as Qualter et al. (2015) suggest. However, we also note that it is still unclear what adaptive cognitive and behavioral patterns might look like. Additionally, in the discussion, we address the lack of adaptive tendencies in our findings, noting that our cross-sectional design may not capture short-term adaptive social responses observed in experience sampling studies (e.g., Reissmann et al., 2021). These revisions provide a clearer contrast between adaptive and maladaptive loneliness and acknowledge methodological limitations.

Introduction

“While Qualter et al. [10] discuss how individuals in this state of hypervigilance can escape these maladaptive tendencies and show more adaptive behaviors with interventions, the question remains unclear what cognitive and behavioral patterns are associated with adaptive loneliness. Nonetheless, this distinction between adaptive and maladaptive loneliness highlights the importance of considering not just the presence of loneliness but its persistence, intensity, and cognitive and behavioral consequences. Understanding what maladaptive and adaptive experiences of loneliness encompass remains a critical area for further research.”

Discussion:

“*However, a major remaining open questions is the scarce research regarding the adaptive nature of loneliness [see for review: 11]. A possible reason for the absence of adaptive tendencies in our results could be the cross-sectional design and the focus on more trait-like loneliness, which does not allow for the examination of situational processes that might be adaptive, even if the overall pattern of loneliness appears maladaptive. For instance, Reissmann et al. [8] used experience sampling methodology (ESM) to track fluctuations in social interactions in response to momentary feelings of loneliness, prompting participants multiple times a day to report their loneliness levels and social interactions. Their findings indicated that loneliness led to both increases and decreases in subsequent social interactions [8].”*

Comment 2:

- Measurement choices: the authors critique the use of the UCLA Loneliness Scale and other measurement tools for their limitations, such as not capturing the intensity or duration of loneliness adequately. However, the manuscript does not provide a clear rationale for the selected measures or how they were specifically adapted for this study. In addition, the choice of cut-off points for categorizing loneliness is somewhat arbitrary and lacks a robust justification.
- Response: Thank you for your feedback. We have clarified the rationale for our selected measures in the methods section, highlighting how each was chosen to capture distinct facets of loneliness (frequency, distress, and chronicity) in line with recent theoretical discussions (e.g., Qualter et al., 2021; Maes & Vanhalst, 2024). We did expand on the rational for the cut-off of 24 months in the method section due to the feedback of the other reviewers. However, we acknowledge that the cut-off points, while based on prior research (e.g., Young, 1982; Reinwarth et al., 2023), are somewhat arbitrary and have noted this as a limitation in the discussion.

Methods:

*“Loneliness chronicity was operationalized by again using the above-mentioned direct single-item question. For participants who reported feeling lonely at least quite often, we asked for how many months this was already the case. We then created a dichotomous variable (1 = lonely and 0 = non-lonely) with a cut-off value of 24 months in accordance with the definition by Young [43], which has been used in previous cross-sectional [44-46] and longitudinal studies [e.g., 47, 48-52].”*

Limitations:

“*Eighth, even though the cut-off points were extracted from previous studies or theories, they remain somewhat arbitrary. Changing these would have also changed the results. Nevertheless, the three methods did not seem to capture the same individuals entirely.”*

Comment 3:

- Sample limitation: the sample is predominantly female (81%) and relatively young (mean age of 31.86 years). While the authors note the limitations of generalizability, they do not adequately explore how these demographic factors might influence the findings. For example, the experiences and expressions of loneliness may differ significantly across genders and age groups.
- Response: Thank you for highlighting this point. We have expanded our discussion of how the predominantly female and relatively young sample limits the generalizability of the findings.

*“Third, the sample was predominantly female (81%), which limits generalizability. While a previous meta-analysis has found that gender differences in loneliness are generally small [94], a newer study reported higher levels of loneliness in men than women [95]. Fourth, our sample was relatively young. While this limits the generalizability of our findings to older or adolescent populations, prior research suggests that key cognitive and behavioral mechanisms underlying loneliness - such as interpretation bias and social avoidance - are relevant across age groups [10, 13]. Nonetheless, future studies should investigate whether the relationships observed in our study differ across developmental stages.”*

Comment 4:

- While the statistical analyses employed, such as ANCOVA and χ2 tests, are appropriate, the manuscript could benefit from a more detailed explanation of why these specific methods were chosen over others. Additionally, the analyses focus on group differences, but they lack a discussion on the potential for mediating or moderating variables that could provide deeper insights into the relationships being examined.
- Response: Thank you for your valuable feedback. We have expanded the methodological section to provide a clearer rationale for our choice of statistical methods. Specifically, we have clarified why χ² analysis was used for assessing the degree of agreement between loneliness classification methods and why ANCOVA was chosen to control for depressive and social anxiety symptoms while isolating the effects of loneliness on cognitive and behavioral variables.

Additionally, we acknowledge the importance of investigating potential mediating and moderating mechanisms in loneliness research. While our study focused on group differences, we have added a discussion on how future research could examine mediators such as cognitive biases (e.g., interpretation bias) and moderators such as self-esteem to better understand the mechanisms that sustain or buffer against loneliness. This addition highlights the need for further studies exploring these pathways, which could enhance theoretical models and inform intervention strategies.

Methods

“*Fourth, we tested the degree of agreement between the three classification methods using χ² analysis, which is appropriate for assessing categorical variable associations. Since loneliness classifications in this study are dichotomous (lonely vs. non-lonely), χ² allows us to determine whether the distributions of participants across methods significantly differ beyond random chance.*”

“*Sixth, we performed three (for each classification method) two-sided ANCOVA to test group differences regarding interpretation bias, rejection sensitivity, social avoidance behavior, distress disclosure, self-esteem, and avoidance goal intensity while controlling for depressive (PHQ-9) and social anxiety symptoms (SIAS-6) by including them as continuous covariates. This approach allowed us to isolate the specific effects of loneliness facets on cognitive and behavioral variables and ensured that observed differences are not solely driven by underlying psychopathology.”*

Discussion

“*While our findings highlight significant cognitive and behavioral differences between lonely and non-lonely individuals, future research could explore potential mediating or moderating mechanisms that shape these relationships. For instance, cognitive biases, such as negative interpretation bias, may mediate the link between loneliness and social withdrawal, reinforcing the cycle of loneliness, as previous cross-sectional studies have suggested [22]. Similarly, self-esteem could serve as a protective factor, potentially buffering against the negative effects of loneliness. Prior research has shown that self-esteem moderates the impact of loneliness on life satisfaction and symptoms of depression and anxiety [88-91]. Examining these mechanisms could provide deeper insights into how loneliness develops and persists, ultimately informing more targeted interventions*.”

Comment 5:

- The results section presents significant differences across groups; however, the discussion lacks a critical interpretation of these findings. For instance, the authors assert that increased loneliness correlates with higher rejection sensitivity and avoidance behaviors, yet they do not address the implications of these relationships adequately. How might these cognitive and behavioral tendencies manifest in real-life social contexts? A more profound exploration of the practical implications of the findings would enhance the relevance of the research.
- Response: Thank you for your thoughtful feedback. We agree that a deeper discussion of the real-life implications of the observed cognitive and behavioral patterns would enhance the manuscript. . We have revised this section in response to previous reviewers' and your comments to provide a more nuanced interpretation of our findings, and have now explicitly elaborated on how these tendencies could influence real-world social dynamics.

*“Lonely individuals exhibited significantly greater interpretation bias, aligning with previous research demonstrating that they tend to interpret ambiguous social situations negatively [15, 16]. This bias, in turn, can reinforce rejection sensitivity, where we found significant group differences. Lonely individuals tend to anticipate rejection, leading them to be more vigilant for social threats [79, 80], which is theorized to promote social withdrawal [10, 12]. Accordingly, our results show that loneliness comes with increased social avoidance behavior. This finding is in line with studies showing that lonely individuals are more likely to engage in behaviors that avoid social interactions [80, 81]. Notably, rejection sensitivity has been shown to predict social avoidance behavior [80] and thus may potentially exacerbate feelings of loneliness. These tendencies may manifest in everyday social settings, such as hesitancy to initiate conversations in group settings, reluctance to seek emotional support, or misinterpreting neutral social cues as rejection. Over time, such patterns could contribute to difficulties in maintaining friendships and hinder the formation of new social bonds.*

*Additionally, our results suggest that loneliness appears to be linked to higher avoidance goal intensity, which stands in line with previous research [82, 83]. This means that lonely individuals seem more motivated to avoid negative outcomes. Similarly, Mund and Neyer [84] found significant associations between loneliness and prevention focus (i.e., avoiding harm in social situations). This increased avoidance motivation might contribute to reduced distress disclosure, which corroborates findings of previous studies [85, 86] and could potentially hinder relationship formation and deepen (subjective) isolation. Finally, the large effect sizes of the group differences observed for self-esteem suggest that self-esteem is a central feature of all three facets of loneliness. Previous research has consistently found a strong link between loneliness and self-esteem [20, 21, 87], indicating that low self-esteem might reinforce loneliness. However, there is also the potential for high self-esteem having a buffering effect on loneliness.”*
